# Deformation Induced Hierarchical Twinning Coupled with Omega Transformation in a Metastable β-Ti Alloy

**DOI:** 10.1038/s41598-018-37865-0

**Published:** 2019-02-04

**Authors:** S. A. Mantri, F. Sun, D. Choudhuri, T. Alam, B. Gwalani, F. Prima, R. Banerjee

**Affiliations:** 1 Department of Mater Science and Engineering, University of North Texas, Denton, TX 76207 USA; 2 PSL Research University, Chimie ParisTech-CNRS, Institut de Recherche de Chimie Paris, 75005 Paris, France

## Abstract

Hierarchical twinning, at multiple length scales, was noted in a metastable body-centered cubic (bcc) β-titanium alloy on tensile deformation. Site-specific characterization within the deformation bands, carried out using EBSD and TEM, revealed {332} <113> type primary *bcc* twins, containing different variants of secondary and tertiary twins, as well as the formation of stress-induced martensite (α”). Within the primary {332} <113> type twin, “destruction” of the prior quenched-in athermal ω phase was observed, while a stress-induced ω phase reforms within the tertiary twins, revealing the intricate nature of coupling between deformation twinning and displacive ω transformation.

## Introduction

The deformation behavior of β-titanium alloys has been closely linked to the chemical stability of the parent β-matrix^1,2^. It has been reported in the past that an increased β-phase stability leads to deformation via slip while a reduced or metastable β-phase is more prone to stress-induced products and/or deformation twinning^3,4^. Unlike other *bcc* alloys wherein the common mode of twinning is {112} <111>_*β*_^5,6^, {332} <113>_*β*_ type of twinning is the more predominant mode in metastable β-Ti alloys^7,8^. Since its first discovery in 1970 by Blackburn and Feeney^9^, many researchers have focused on understanding the formation of these kind of twins, owing to their strong influence on the mechanical behavior of these alloys. Hanada *et al*. suggested that the formation of {332} <113>_*β*_ type of twinning is closely associated with alloys which form an athermal ω on quenching from the β phase field^1^. This has been associated with the shuffle mechanism for twin formation, suggested by Crocker^10,11^, which is similar to the shuffle mechanism for the ω formation^8^. Since then a lot of research has focused on understanding the formation of this unique type of twins. Tobe *et al*.^12^, Lai *et al*.^13^, and Castany *et al*.^14^ have all proposed different mechanisms regarding the formation of the {332} <113> type of twins. While Tobe *et al*. put it down to the lattice instabilities observed in the metastable β-Ti alloys, Lai and Castany have attributed the formation of {332} <113> twins with the stress induced martensite (SIM) phase. Recently Chen has also shown the transitional structure of the twin boundaries using high-resolution TEM images^15^.

Interestingly, it should be noted that the formation of this unique type of twin is often associated with systems which also form diffusionless transformation products, such as athermal omega (ω_ath_) and SIM, though the relationship between the two is still not very well understood. While there have been previous reports on the interaction between slip and ω_ath_, leading to the formation of ω-free channels in the β matrix^3,16–19^, there have been no previous reports on the interaction between deformation twins and the prior existing ω_ath_. The present paper focuses on the hierarchical nature of deformation twinning and its interaction with prior existing ω_ath_ precipitates, during quasi-static tensile deformation of a metastable β-Ti alloy.

## Results

**Figure 1:**
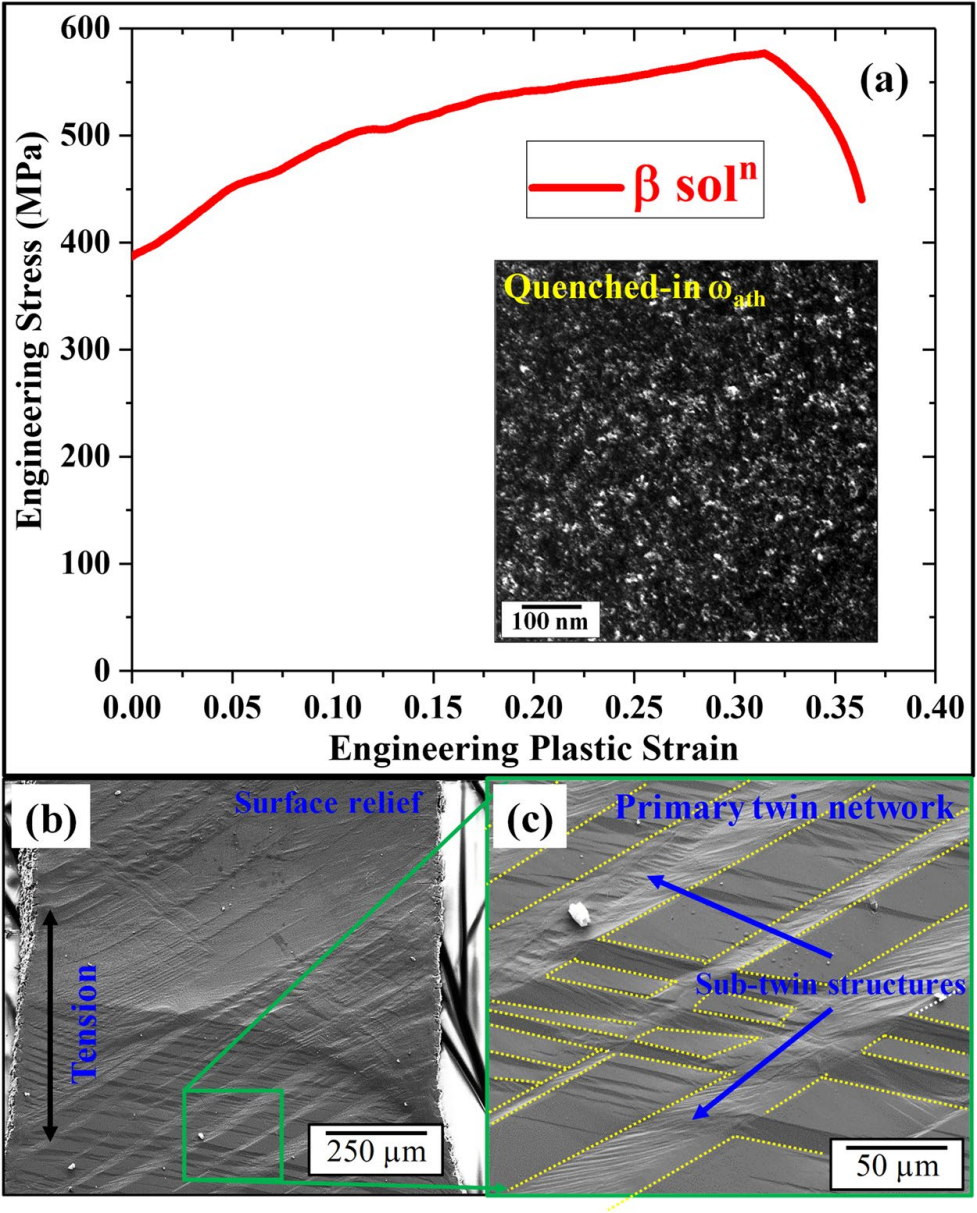
(**a**) Stress-strain plot of b-soln condition with the inset showing the starting microstructure, (**b**,**c**) SEM images after deformation showing the surface relief and twins.

The mechanical behavior of the ST sample, plotted as an engineering stress vs strain plot, is shown in Fig. 1(a). The starting microstructure, shown as an inset, contains athermal ω precipitates within the parent β matrix. The formation of the ωath takes place congruently after the quenching from above the β-transus temperature (from the single β phase field). We have shown in past studies, via Atom Probe Tomography that there is no compositional partitioning between the β matrix and the ω_ath_ precipitates^3,20^. Additionally, based on TEM dark-field images, the ω_ath_ was found to be homogenously distributed throughout the β-matrix and these particles have a size range of about 2–5 nm. The stress-strain plot shown in Fig. 1(a), indicates a substantial degree of strain hardening leading to a UTS value of ~550 MPa starting from a yield of ~400 MPa, and tensile ductility of 35%. Figure 1(b,c) shows SEM backscattered images, at different length scales, of a pre-polished sample following deformation to failure. Surface relief can be clearly observed in the system due to the tensile deformation and these images also reveal twinning occurring at multiple length scales across the sample. Within the primary twin network, a secondary sub-twin structure is noted.

**Figure 2:**
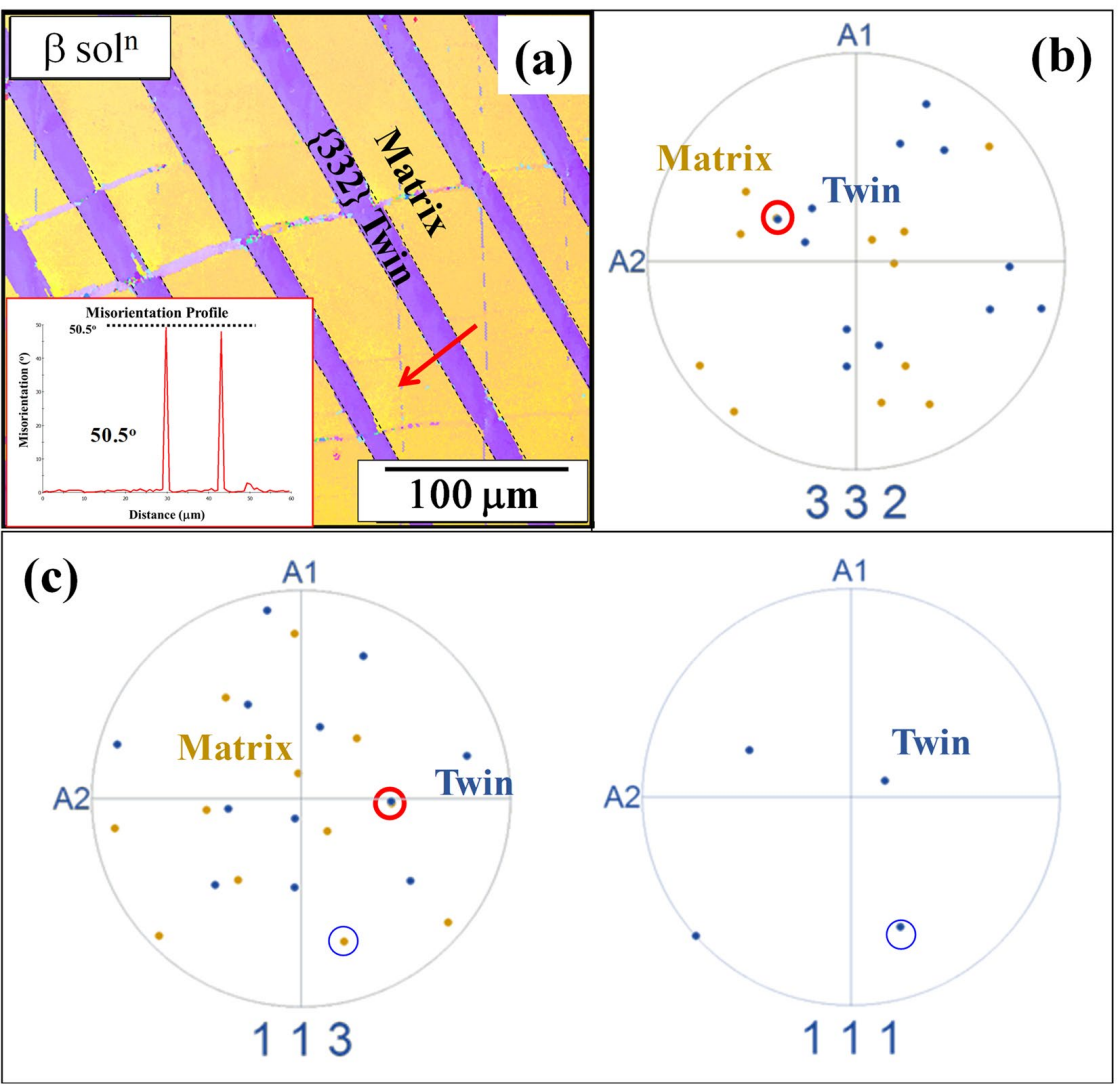
(**a**) EBSD map showing the {332} <113> twin with inset showing a misorientation profile of 50.5°, (**b**,**c**) composite discrete plots showing the common planes for both {332} and {113} plots.

Following the initial analysis using SEM, EBSD was performed on the sample to further understand the nature of twins. Figure 2(a) shows the EBSD IPF map of the sample after deformation to failure. The EBSD was done away from the fracture surface to get a good confidence index during the EBSD analysis. The inset in this figure shows the point to point misorientation profile across the matrix and twin, as pointed with the red arrow. The misorientation profile across the β matrix and twin was noted to be around ~50.5° which, based on previous literature reports, points to the twin being of {332} <113> type. The discrete pole figure plots in Fig. 2(b), with a superimposition of the {332} planes of the matrix and twin show the common planes of the matrix and twin marked with circles. Similar arrangement of the {113} planes of the matrix and twin is shown in Fig. 2(c). These discrete plots provide further confirmation regarding the type of twin. Additionally, it should be noted that there is a {113} pole (different from the common twin pole) of the matrix aligning approximately parallel to a {111} plane of the twin, also shown in Fig. 2(c). A more detailed analysis of the deformation microstructure, and products within the exact same primary deformation band, has been carried out via TEM on a site-specific sample that was extracted from this band using the dual-beam FIB/SEM (shown as a Supplementary Figure).

**Figure 3:**
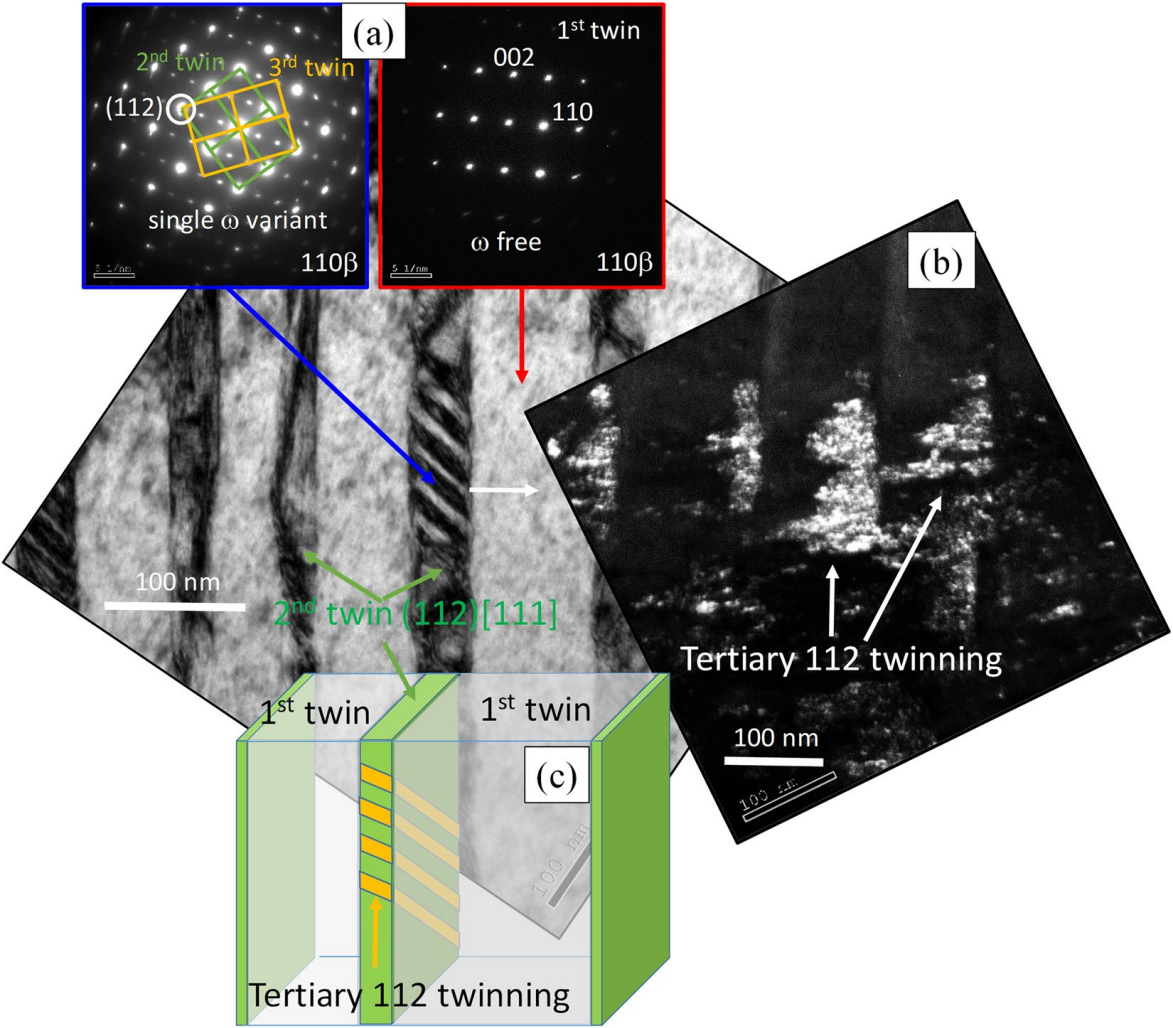
(**a**) Low magnification BFTEM image of FIB foil showing the primary {332} <113> twinning and the β-matrix. Inset shows SADP taken along {110}β matrix, indicating the presence of ω. (**b**) High magnification image of the *band* separating the matrix and the primary twins. SADP taken from within the band and the interface of *band*/matrix are shown in purple and green respectively (schematic illustration of the DP is also shown here). (**c**,**d**) BFTEM images showing the secondary and tertiary twinning within the primary twinning.

Low magnification bright-field TEM (BFTEM) image of the FIB foil, shown in Fig. 3(a) reveals the presence of multiple features across the sample. A thin plate, indexed as band 1 for convenience, separating the β matrix and the β twin is revealed. A higher magnification image of this band is shown as an inset (Fig. 3(b)) and will be discussed in detail in further sections. The selected area diffraction pattern (SADP) taken from the matrix (white circle) confirms the presence of the ω phase, shown as the starting the microstructure in Fig. 1(a). It should be noted here that both the variants of the ω phase are present. The next feature of interest observed within the sample is the presence of the *secondary* twinning within the primary β-twin. A higher magnification image of this image is shown in the Fig. 3(c). The hierarchical nature of the twins continues in that, within these *secondary twins*, the presence of *tertiary* twins is also noted. The nature of the secondary twins is not yet fully understood, but previous reports by Sun *et al*. have indicated that these twins could either be another variant of {332} <113> type of twins or the more common {112} <111> type of twins^21^. The increase in the strength and improved ductility could be attributed to the presence of these hierarchical deformation twins. Wei *et al*. have previously addressed the importance of a hierarchical structure in improving the mechanical behavior of alloys, wherein by having a hierarchical nano-twin structure an improved strength and ductility was noted in steels^22^. Figure 3 shows that the secondary and tertiary twins form in the regions between the primary twins and secondary twins, respectively. This implies that the formation of these twins is blocked by their precursors, which leads to the strain hardening effect noted in this alloy. Along with the twin/twin interactions, there is a high probability of dislocation/twin interaction, which also plays a role in the increased strain hardening effects^23^. Lai, Castany, and Chen have all previously reported that the SIM (α”) plays a role in the formation of {332} <113> type of β-twin. The nature of the band/plate, shown in Fig. 3(a,b) on first viewing could be misconstrued as to being this SIM (α”) plate. But, on further examination, SADP from the band (purple circle) reveals that the band is in fact still *bcc*-β with secondary {112} <111> β type twins (transversal fine laths). The SADP taken from the interface of the β matrix and the band (shown in the green circle) confirms the presence of the primary β (light blue), SIM α” (magenta) and the β band 1 (green). The schematic diagram showing the DP helps us better understand this SADP. Three distinct features can be clearly observed: (i) β matrix with the two ω variants, (ii) β band 1 with no ω variants but secondary twins, (iii) trace of SIM (α”). While the presence of (i) and (ii) is to be expected, the presence of the SIM is rather peculiar. Castany *et al*. have shown that during the deformation process, the formation of SIM precedes the {332} <113> type of twinning and once the twinning starts, the presence of SIM is no longer noted. Accordingly, we believe the reflections seen here are remnants of the SIM formed during the early stages of deformation. According to the orientation relationships among three of them, it is observed that SIM (α”) and β matrix respects the classic relationship via martensitic transformation (112) β matrix//(011) SIM α”. However, none of known twinning relationships could be found neither between β matrix and β band 1 nor between primary 332T and β band 1. Interestingly, the DP of β band 1 shared a common (112) β spot with SIM at (0–11) α”, a signature means β band 1 and SIM also respects the classic martensitic relationship. Considering that this is the only crystallographic link of band 1 to its neighbors, i.e. the β matrix on the right and primary 332T on the other side, it would be reasonable to assume that the band 1 could probably be the product of reversion of SIM α”. The SIM’s product during unloading, similar to the findings of Castany *et al*., could be altered from its parent β phase according to the local strain accommodation. Nevertheless, the band 1 found here was not in 332 twinning relationship to the parent β phase, i.e. the β matrix, which is inconsistent to Castany *et al*. reports.

**Figure 4:**
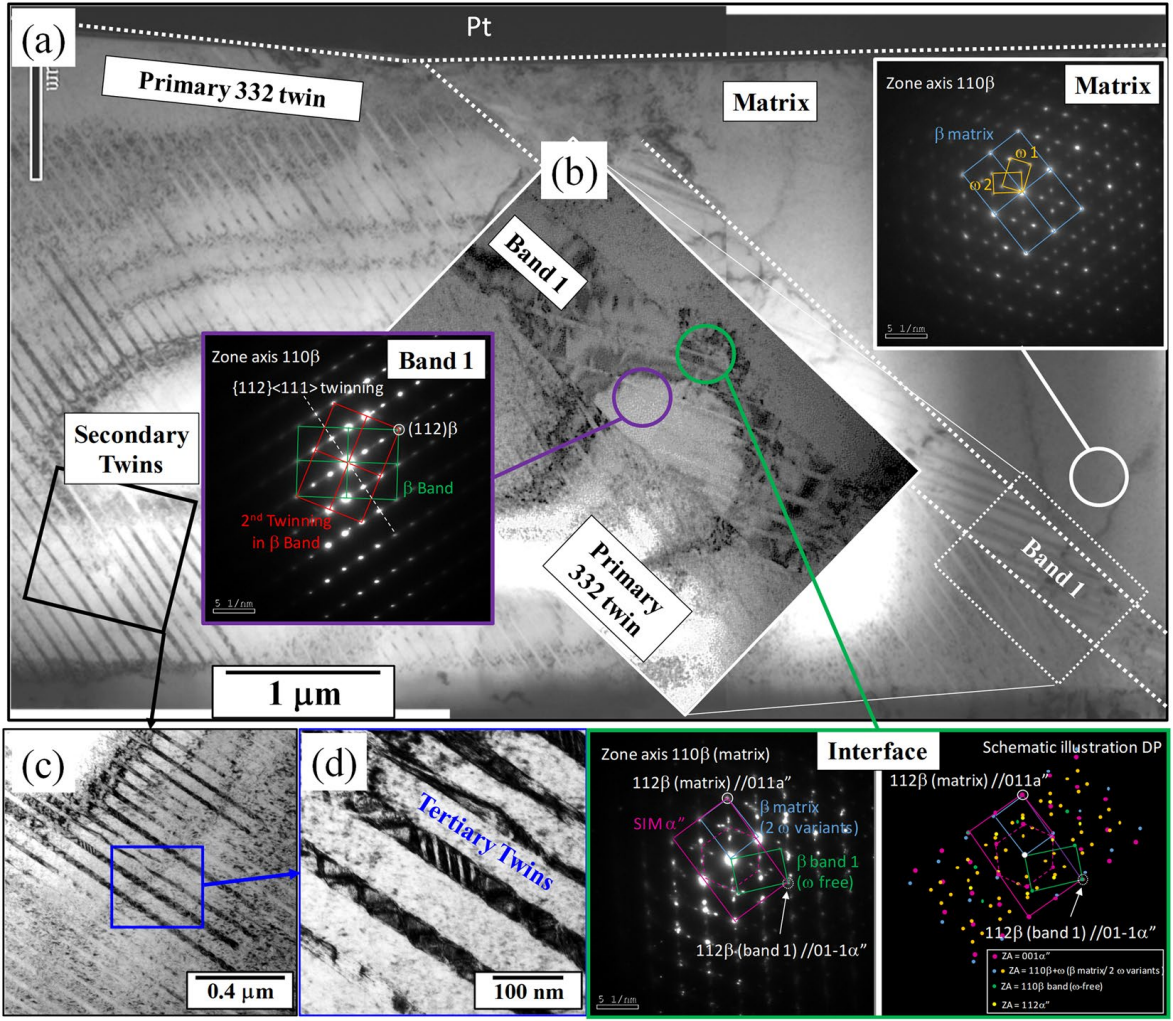
(**a**) SADP obtained from two different regions within the secondary {112} [111] twinning shows the selective omega variant formation and the destruction of omega is shown via a lack of the omega reflections. (**b**) DFTEM obtained from selecting the reflections shown in Fig. 4(a) shows the presence of omega particles next to tertiary twinning, (**c**) schematic illustration of the BFTEM image shown in (**a**).

The presence of ω within the matrix of this sample has already been established earlier with the dark-field TEM image shown as an inset in Fig. 1(a), and the [011]β zone axis SAD patterns shown in Fig. 3(a). It is therefore of interest when we look at the SADP obtained from the β twinned region, in Fig. 4(a). While the two patterns shown here are obtained from the *primary* twin, the SADP in blue is from the *tertiary* twins and the SADP in red is from the *matrix* of the *primary* twin. The lack of additional reflections at 1/3 and 2/3 {112}β locations, corresponding to the ω phase, establishes the absence of the pre-existing quenched-in athermal ω precipitates within the *primary twin matrix*. From these, it appears that on the formation of the *primary* twin, the shuffle within the precipitates of the ω phase has been reversed to that of the β structure. Previous reports in the literature have proposed that during the deformation of a β matrix containing ω_ath_, a ω free channel is created due to the shearing of the ω precipitates by the dislocations. This has been attributed to motion of perfect<111> β dislocations on {112}β slip planes^17^. A similar mechanism is likely to be operative in the present case, wherein, during the formation of the *primary* twin, the dislocations shear through the ω_ath_ phase and transform these precipitates back into the structure of the β matrix (the *primary* twin). An investigation of the interaction of deformation twins with pre-existing athermal ω precipitates will be the subject of future work. [011]β zone axis SAD patterns recorded from regions near the *tertiary* twins (marked with the blue box in Fig. 4(a)) reveals the presence of only one set of 1/3 and 2/3 {112}β reflections. A relatively strong intensity can be clearly observed, indicating the preferential formation of one particular ω variant. This is likely to be a stress-induced ω phase forming near the *tertiary* twins within the *primary* twin plate. While such stress-induced ω formation has been previously reported in similar *bcc* systems^7,21,24,25^, the exact location and morphology of these ω precipitates within the deformed microstructure are not well understood. A DFTEM image, recorded from one set of 1/3 and 2/3 {112}β reflections in the SAD pattern shown in Fig. 4(a), is shown in Fig. 4(b). The contrast within this dark-field image indicates that the stress-induced ω precipitates at the interface between the *tertiary* twins and the matrix (primary twin). While this has been previously reported in titanium alloys^2,25^, more extensive studies have been previously reported on shock-induced impact deformation in tantalum and tungsten alloys, providing an insight into this phenomenon^24,26^. The mechanism of stress-induced ω formation has been attributed to the glide of the 1/3[111], 1/6 [111], and 1/12 [111] partial dislocations, which disassociate from the perfect 1/2 [111] dislocation, in the *bcc* structure^26^. Figure 4(c) is a schematic showing the microstructure with the hierarchical twinning observed in this system.

**Figure 5:**
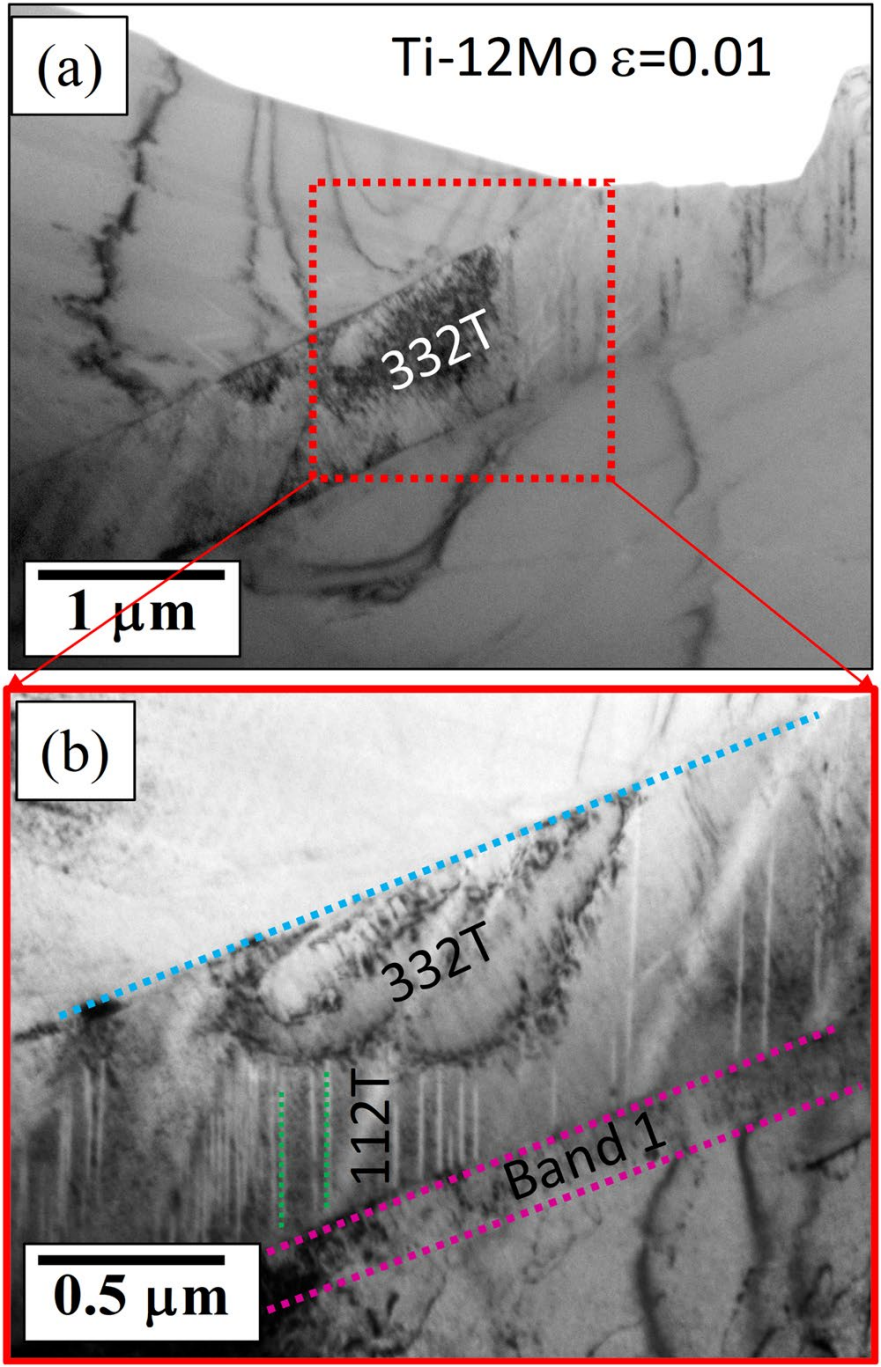
(**a**) Low magnification BFTEM after 1% strain showing the presence of {332} <113> twin (**b**) High magnification image of the same area showing the “band” and also the presence of {112} <111> twins within the primary {332} <113> twin.

To better understand the deformation behavior at early stages, another Ti-12Mo sample was strained to 1% and analyzed in the TEM for investigating the deformation structure (Fig. 5). Similar to the fully strained sample, the TEM sample prepared from the 1% strained material, was made in such a way that half of it covered the β-matrix and the other half contained the twin. Figure 5 shows the BFTEM image of the 1% strained sample at two different magnifications; clearly showing the presence of a coarse primary {332} <113> twin, together with an adjacent “*band*” similar to the one observed in Fig. 3 (strained to failure sample). This “*band*” is believed to be the stress induced martensitic (SIM) plate, which after deformation to failure (Fig. 3), relaxes and transforms back to another β-band with a different orientation as compared to both the matrix as well as the primary twin. The presence of {112} <111> secondary twins are also noted within the primary {332} <113> twin, but the volume fraction and the size of these are much less as compared to the deformed to failure sample as seen in Fig. 3.

## Discussion

**Figure 6:**
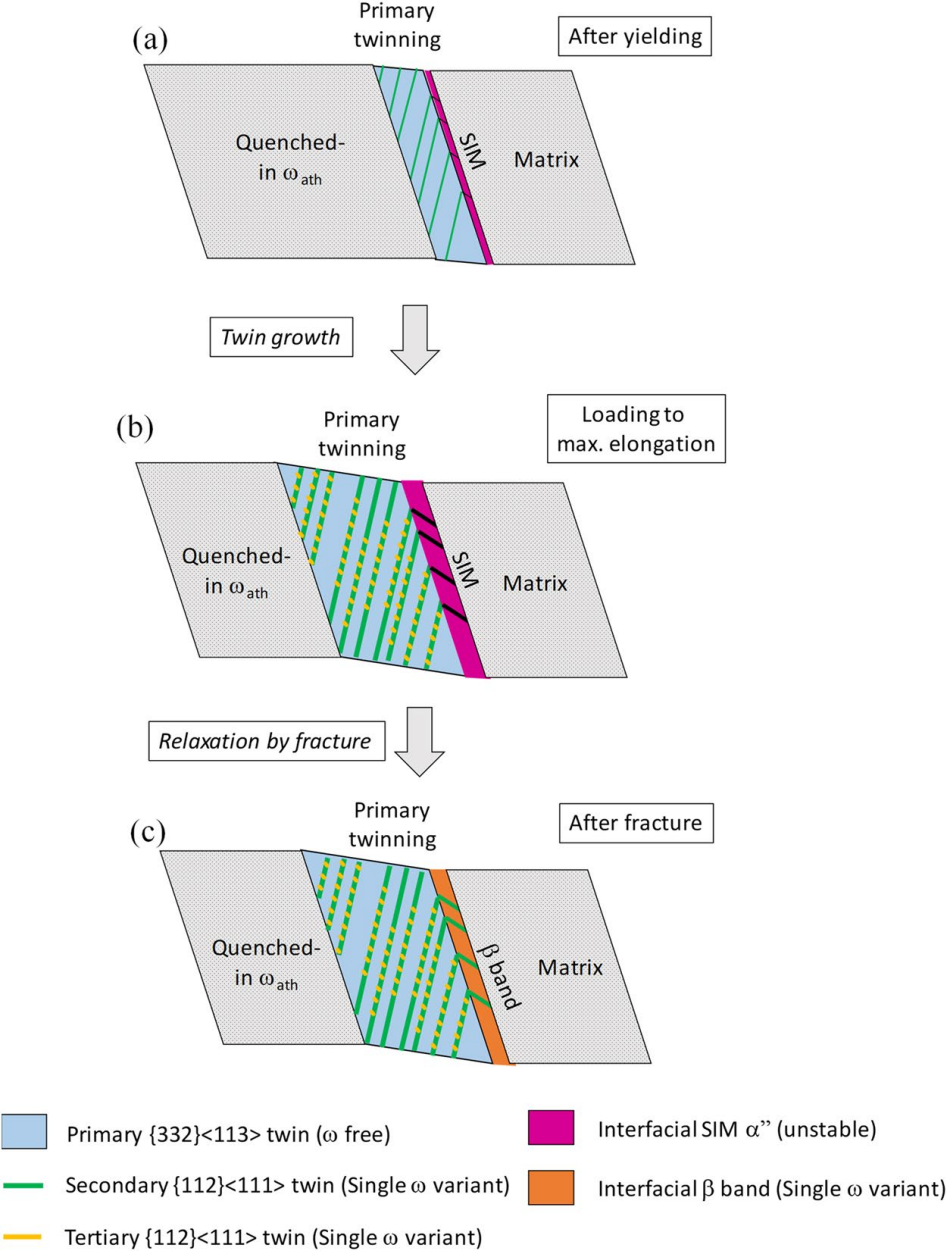
Schematic showing the sequence of the formation of deformation products during the tensile testing.

While the sequence of events cannot be conclusively determined from this post-mortem investigation, based on the past literature and the experimental observations presented here, Fig. 6 shows the most likely order of events during the tensile deformation of the Ti-12Mo sample.

1. With increasing stress, the 332 twinning and SIM α” plate forms simultaneously, and these martensitic plates nucleate at the boundary of the primary {332} <113> twin plates. Within the twin plate, the restructuring of the ω phase back to parent *bcc* takes place. The early stage formation of *secondary* twins within the *primary* twin are also noted.
2. After loading to the maximum elongation, there is an overall increase in the width of both the primary twin and the SIM plate. The SIM plate is characterized by internal linear defects or twinning to accommodate the secondary twinning in its 332T neighbor. Due to increase in the loading, formation of *tertiary* twins within the primary twin band follows. The formation of tertiary twins ({112} <111> type) leads to the formation of stress-induced ω within these plates.
3. After the fracture, upon relaxation, the SIM transforms back into another β phase other than β matrix, with secondary twinning within and trace remnants of the SIM plate. The relaxation does not seem to affect the primary twin band or the subsequent twinning in anyway.

## Conclusion

To summarize, coupling SEM-EBSD studies with site-specific TEM characterization on a tensile tested Ti-12Mo alloy sample, leads to some novel insights into the complex hierarchical deformation processes operative in these metastable β titanium alloys, over multiple length scales. Formation of two different types of twins namely {332} <113> and {112} <111> are seen along with the stress-induced formation of both the α” and ω phases. A hypothesis based on the past literature and present experimental results explains the sequence of the microstructural changes leading to an increased strain-hardening effect.

## Methods

The binary Ti-12 wt.%Mo (hereafter referred to as Ti-12Mo) alloy was fabricated by vacuum arc melting using pure Ti and Mo. Following this, they were solution-treated (ST) at 900 °C for 30 min and subsequently water-quenched. Microstructural characterization was performed using scanning electron microscopy (SEM), FEI NovaNano SEM230, and transmission electron microscopy (TEM), FEI Tecnai F20-FEG TEM operating at 200 kV. As the EBSD analysis was carried out after deformation to failure, the analyzed regions were intentionally selected away from the fracture surface, more towards the grip section, in order to get a better indexing in EBSD, and a schematic showing the location from which the analysis was done is shown in the Supplementary Figure. Site-specific TEM foils were prepared via a FEI Nova NanoLab 200™ focused ion beam (FIB). The sample was made in such a way that half the foil was from the β-matrix and the other half from the β-twin. The location of this has also been shown in the aforementioned schematic. Dog-bone shaped tensile specimens of gage length ~5 mm, width ~1 mm and thickness ~0.8 mm were used for the mechanical testing, which was done under uniaxial tension at a strain rate of 10^−3^ s^−1^ at 25 °C. Details of the setup for the mechanical testing are presented elsewhere.

## Supplementary information

Supplementary Information
