# Supplementary material for: Deformation Induced Hierarchical Twinning Coupled with Omega Transformation in a Metastable β-Ti Alloy
Source: Sci Rep. 2019 Feb 4;9:1334. doi: 10.1038/s41598-018-37865-0 (PMC6362253; doi:10.1038/s41598-018-37865-0)

**Deformation Induced Hierarchical Twinning Coupled with Omega Transformation in a Metastable β-Ti Alloy**

S.A. Mantri^1^, F. Sun^2^, D. Choudhuri^1^, T. Alam^1^, B. Gwalani^1^,
F. Prima^2^, R. Banerjee^1,^ *


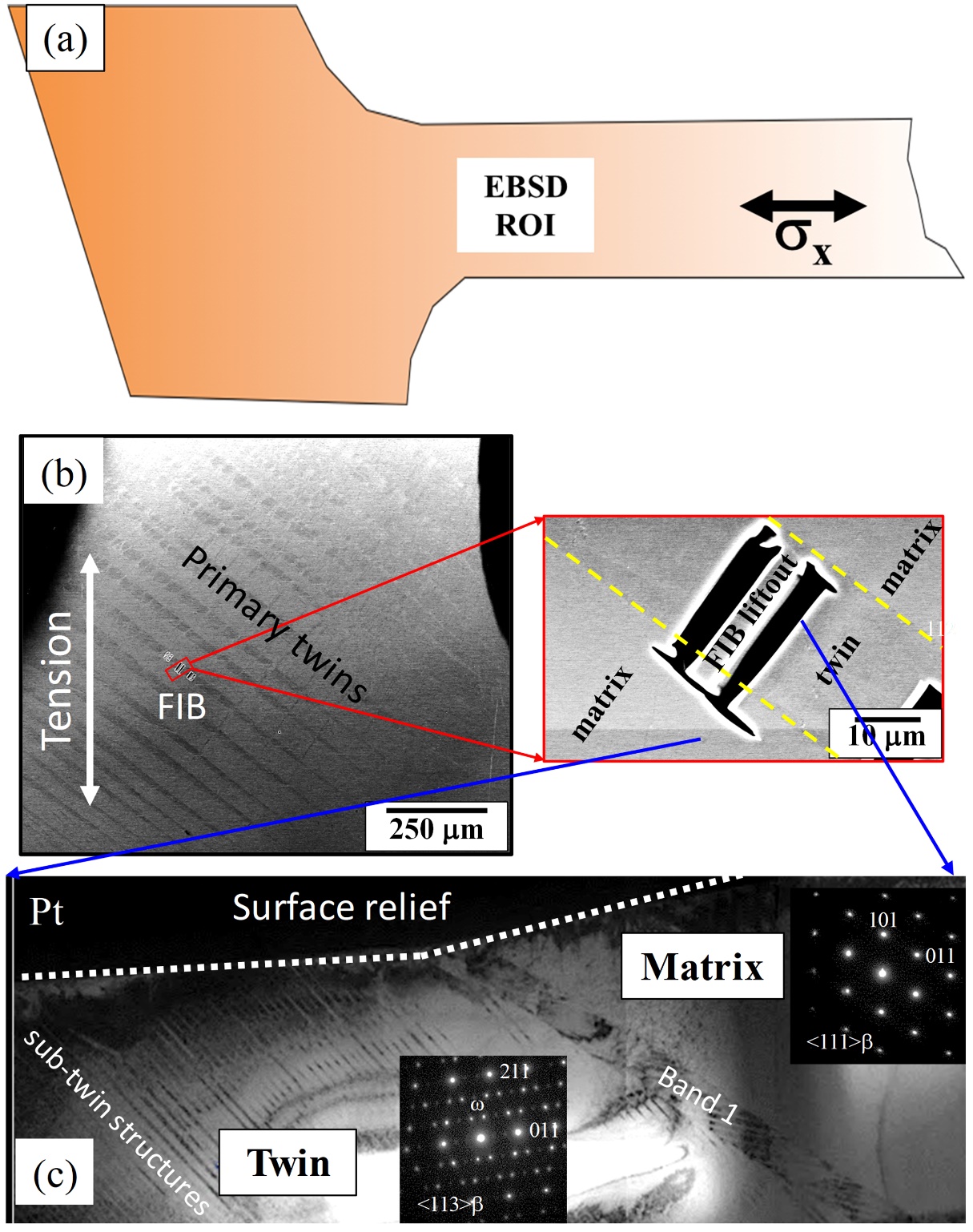

Supplement: Supplementary file 1 — Supplementary Information [file 41598_2018_37865_MOESM1_ESM.docx]
